# A grassroots approach for greener education: An example of a medical student-driven planetary health curriculum

**DOI:** 10.3389/fpubh.2022.1013880

**Published:** 2022-09-26

**Authors:** Allison Navarrete-Welton, Jane J. Chen, Blaire Byg, Kanika Malani, Martin L. Li, Kyle Denison Martin, Sarita Warrier

**Affiliations:** ^1^Warren Alpert Medical School of Brown University, Providence, RI, United States; ^2^Department of Emergency Medicine, Kent Hospital, Warwick, RI, United States

**Keywords:** planetary health, medical education, climate change, medical waste, curriculum development, environment, public health

## Abstract

Given the widespread impacts of climate change and environmental degradation on human health, medical schools have been under increasing pressure to provide comprehensive planetary health education to their students. However, the logistics of integrating such a wide-ranging and multi-faceted topic into existing medical curricula can be daunting. In this article, we present the Warren Alpert Medical School of Brown University as an example of a student-driven, bottom-up approach to the development of a planetary health education program. In 2020, student advocacy led to the creation of a Planetary Health Task Force composed of medical students, faculty, and administrators as well as Brown Environmental Sciences faculty. Since that time, the task force has orchestrated a wide range of planetary health initiatives, including interventions targeted to the entire student body as well as opportunities catering to a subset of highly interested students who wish to engage more deeply with planetary health. The success of the task force stems from several factors, including the framing of planetary health learning objectives as concordant with the established educational priorities of the Medical School's competency-based curriculum known as the Nine Abilities, respecting limitations on curricular space, and making planetary health education relevant to local environmental and hospital issues.

## Introduction

With the rising urgency of climate change, medical schools cannot ignore the impact of environmental problems on students and patients. In recent years, increased attention has been focused on how to prepare future doctors to address the health impacts of climate change, biodiversity loss, and environmental degradation. However, the medical education community has not settled on a unified approach to planetary health (PH) education. Implementing a PH curriculum is a particularly daunting challenge given the novelty of the field for many doctors and the overwhelming number of topics encompassed within (1). The magnitude of this challenge is significant, as revealed by surveys completed by the International Federation of Medical Student Associations in 2019–20 that found that only 14.7% of medical schools globally included climate change and health within the curriculum and only 11% incorporated education about the health impacts of air pollution (2).

Multiple groups have proposed overarching principles to guide the creation of PH curricula from a top-down perspective. For instance, in 2019 the Planetary Health Alliance convened a task force to develop a framework for PH education intended “to move beyond a prescriptive list of competencies”. The task force proposed five foundational domains for PH education (equity and social justice, interconnection within nature, movement building and systems change, systems thinking and complexity, and the Anthropocene and health) that they conceptualized as being embedded within learning priorities guided by local and global conditions (3). Alternatively, Maxwell and Blashki proposed using a triad of outcomes to guide curriculum development: climate change preparedness (involving clinical management of climate-related illness and knowledge of how to provide healthcare sustainably), depth of education (using climate change as an illustrative example to deepen the existing knowledge and skills of medical graduates), and breadth of education (public and eco-health literacy) (4). Separately, an international workshop used a collaborative approach to identify five domains meant to provide an overarching framework for the development of specific learning objectives. The domains included eco-medical literacy and clinical preparedness, proficiency in promoting eco-health literacy both among patients and at the community level, education in the delivery of sustainable systems, and incorporating sustainability as an element of medical professionalism (5).

In other cases, PH education has evolved spontaneously in response to student advocacy and concerns. Students at the Florida International University Herbert Wertheim College of Medicine built on the impacts of environmental degradation that they observed during community service to develop a series of slides about planetary health topics that were inserted into existing lectures (6). At Emory University, two students began by organizing a lunch panel discussion on climate and health and, after the lunch panel garnered a surprising amount of interest, then harnessed the enthusiasm of the student body to develop a proposal for incorporating planetary health topics into the medical school curriculum. Their proposal was accepted by Emory's Executive Curriculum Committee with plans to implement the changes for the class of 2024 (6, 7). The creation of the Planetary Health Report Card by students at University of California, San Francisco School of Medicine and the subsequent Planetary Health Report Card Conference held online in October 2021 provided an important venue for medical students at different schools to learn from each other's experiences and gain advocacy skills (8).

**Figure 1:**
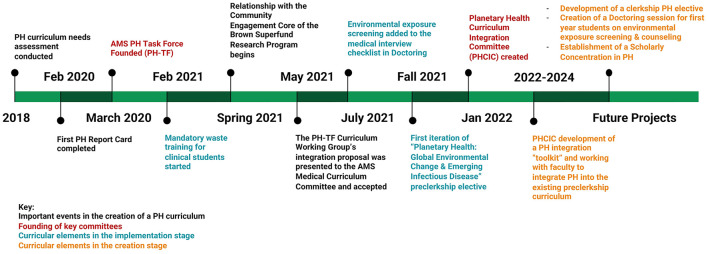
Timeline of planetary health educational initiatives at the Warren Alpert Medical School of Brown University.

Here we present the Warren Alpert Medical School of Brown University as an example of a student-driven, bottom-up approach that has led to the development of a longitudinal PH education program integrated into existing pedagogical priorities. The earliest efforts to expand PH education at the Medical School were disparate initiatives organized and led by students. In 2020, the efforts gained significant momentum when the administration sanctioned the creation of a task force dedicated to improving PH education. Composed of medical students, faculty, and administrators, the Planetary Health Task Force (PH-TF) has focused on both educating the entire student body about PH as well as creating opportunities for highly interested students to engage more deeply with these issues. In this article, we describe the student PH advocacy that led up to the creation of the task force, the curricular changes implemented to date, and the task force's ongoing work to improve PH education (Figure 1). Importantly, we focus on the role of the PH-TF in establishing student-identified PH educational priorities within the Medical School's existing pedagogical framework, known as the Nine Abilities.

To our knowledge, the PH-TF has led to one of the broadest and most successful efforts to date to integrate PH education across all 4 years of medical school in a variety of forms. Although we recognize that each medical school will need to individualize their own PH curriculum, we hope our experiences can be a template for how to incorporate PH into medical education.

## Frameworks

The Medical School's PH curriculum was developed within the existing framework of the Medical School's pedagogical priorities. Since 1996 and most recently revised in 2021, the Warren Alpert Medical School has followed a competency-based curriculum which seeks to define the qualities, abilities, and knowledge that all students should have upon graduating, known as the Nine Abilities. These abilities are: (1) effective communication, (2) basic clinical skills, (3) using basic science in the practice of medicine, (4) diagnosis, prevention, and treatment, (5) lifelong learning, (6) professionalism, (7) health equity and racial justice, (8) moral reasoning and clinical ethics, and (9) clinical decision making. The competencies set prior to the 2021 revision have been described previously in (9, 10).

The PH-TF served as the key vehicle for enacting PH curricular change within the guidelines set by the Nine Abilities. The priorities for PH curricular change were selected in part based on the pre-existing student-driven initiatives that led to the creation of the PH-TF as well as the expertise of PH-TF faculty members in specific content areas. To ensure sufficient scope, the PH-TF consulted previously published frameworks for PH education including the PH learning objectives from the “Climate and Health Key Competencies for Health Professions Students” from the Global Consortium on Climate and Health Education (GCCHE) (11). This approach has enabled the PH-TF's efforts to emphasize the local priorities and interests of our community while avoiding unintentional omissions of crucial PH topics identified from national or international viewpoints.

### Origins of the planetary health task force: Student-driven initiatives and the planetary health report card

Prior to the formation of the PH-TF, medical students led a variety of sporadic sustainability initiatives. On the medical school campus, student advocacy in 2019 resulted in the addition of composting and single-stream recycling. The Student Senate began requiring student group leaders to view a presentation on sustainable event-hosting and commit to following the guidelines. Given the intrinsic connection of PH to community impacts, interested students also frequently engaged with local environmental justice organizations in Rhode Island (RI). These included the Brown Agriculture Nutrition and Community Health program, a collaboration between the Brown Department of Family Medicine and a local elementary school that seeks to address disparities in access to green spaces, nutrition, and health education, as well as political advocacy with grassroots organizations such as Renew RI and Sunrise PVD, tours of the local landfill, and volunteer trips to harvest leftover crops for food banks.

In 2018, a student-led needs assessment drew attention to gaps in the Medical School curriculum regarding PH. The survey, developed by a medical student after consultation with Medical School faculty and Rhode Island Department of Health experts, was sent to all 1st-year medical students (*n* = 144) and achieved a response rate of 50.7% (*n* = 73). This survey found that 95% of first-year medical students agreed that it is important for medical providers to know about the health impacts of climate change, but only 6.8% of students felt that the Medical School provided sufficient education on climate change and health. Furthermore, only 9.6% felt confident discussing health impacts of climate change with patients and only 6.8% felt they knew ways to mitigate the health impacts of climate change (12).

While the needs assessment revealed important shortcomings in PH education, the key event that led to the consolidation of student efforts with the backing of the Medical School administration was the 2020 Planetary Health Report Card (PHRC). The PHRC is a student-driven, metric-based initiative run by the national organization Medical Students for a Sustainable Future. Its goal is to inspire PH and sustainable healthcare education engagement in medical schools across the globe. The PHRC evaluates the performance of medical schools with respect to PH using five metrics including curriculum, research, community outreach, support for student-led initiatives, and campus sustainability (8). In response to the B- grade that the Medical School received in the 2020 PHRC, the administration created the PH-TF.

### Structure of the planetary health task force

The task force includes medical students, faculty, and administrators as well as Brown University Environmental Sciences faculty. The PH-TF is structured as two working groups focused on community engagement and curriculum development, which were the weakest areas according to the Planetary Health Report Card. While the community engagement working group has had difficulty making progress due to COVID-related disruptions affecting local environmental organizations, the curriculum working group has achieved significant results over the past 2 years.

The entire curriculum working group meets on an *ad-hoc* basis ~5 times each year. Subcommittees in charge of implementing specific initiatives meet separately, with varying frequencies.

Curriculum proposals generated by the curriculum working group that affect the entire student body are presented to the Medical Curriculum Committee (MCC) of the Medical School. If the MCC approves a proposal, proposal implementation is led by PH-TF member(s) with the greatest level of interest and expertise in the specific proposal. Depending on the scope of the proposal, this has at times required the creation of a sub-committee, such as the Curriculum Integration Committee described below in Section Integrating planetary health into pre-clerkship material. If the proposal is an initiative targeted to a subset of students interested in PH rather than the entire study body, such as an elective course, it does not necessarily need to be presented to the MCC. When proposals require the involvement of faculty outside the PH-TF, the PH-TF member(s) leading the initiative contact and coordinate with relevant faculty and course directors. To date, the PH-TF has found the faculty who lead the Medical School's pre-clerkship and clerkship courses to be very supportive of the PH-TF's goals and proposals.

### Development of the planetary health core competencies through national and institutional frameworks

Tasked with incorporating a longitudinal PH education into the medical school curriculum, the PH-TF first identified a set of core PH-related skills and knowledge that all students should possess upon graduation. These were adopted from the “Climate and Health Key Competencies for Health Professions Students” from the Global Consortium on Climate and Health Education (GCCHE) to fit the Medical School curriculum (11).

One of the primary strategies for tailoring the GCCHE competencies to the curriculum was to situate the PH competencies within the pre-existing framework of the Medical School's Nine Abilities.

To demonstrate why PH education should be incorporated into the medical school curriculum, the PH-TF emphasized how the objectives of the PH curriculum furthered seven of The Nine Abilities (Table 1). Although different medical schools have different educational priorities, framing the goals of a PH education within preexisting curricular objectives emphasizes the relevance and importance of PH knowledge for future clinicians. It also highlights how PH can be efficiently woven into existing curricula rather than requiring the addition of a new and distinct subject area.

**Table 1:** Planetary health core competencies aligned with guiding educational principles.

**The nine abilities of the medical school**	**Planetary health core competencies**
Ability 1: Effective communication	Be able to effectively communicate with patients about the health impacts of climate change, strategies to prevent those risks, and the concept of health co-benefits of action. Demonstrate effective communication with stakeholders about climate and health topics and work collaboratively and across disciplines on climate and health issues.
Ability 2: Basic clinical skills	Learn to take an environmental history
Ability 3: Using basic science in the practice of medicine	Define climate drivers (both natural and human-caused), weather, climate change, and climate variability. Identify the health impacts of climate change and other human-driven disruptions to our natural environment.
Ability 4: Diagnosis, prevention, and treatment	Apply knowledge of the connection between habitat and biodiversity loss and infectious diseases. Apply knowledge of climate and health to clinical care of patients. Understand how to identify, prevent, and treat commonly seen health related impacts of climate change, i.e., heat stroke, asthma exacerbations, acute kidney injuries, adverse birth outcomes.
Ability 7: Health equity and racial justice	Recognize the disproportionate impact of climate change on communities of color and explain pre-existing and future health disparities rooted in environmental racism, especially in Rhode Island. Apply climate and health knowledge to improve decisions about public health services, and adapt and improve population health. Describe ways medical students and health professionals can engage in institutional, community, and political advocacy around planetary health. Describe ways that healthcare professionals and facilities can prepare for and respond to climate-related health risks.
Ability 8: Moral reasoning and clinical ethics	Appreciate the role of the healthcare industry in contributing to climate change and identify ways healthcare providers can reduce/mitigate waste and carbon emissions.

The planetary health core competencies adopted from the “Climate and Health Key Competencies for Health Professions Students” from the Global Consortium on Climate and Health Education were able to be aligned with seven of the “Nine Abilities” that guide the Medical School curriculum.

## Learning environment, objectives, and format

In line with the PH curriculum's core competencies, the interventions undertaken by the PH-TF span a diverse range of learning environments and formats from traditional core classroom education to an elective course, student-directed research, and extracurricular community engagement opportunities. These diverse initiatives can be categorized into two groups: first, the interventions targeted to include the entire student body and second, the programs designed for the subset of students who desire deeper engagement with planetary health. The format and learning objectives of the specific initiatives are detailed here.

### Student body-wide interventions undertaken by the planetary health task force

#### Integrating planetary health into pre-clerkship material

Prior to the formation of the PH-TF, isolated lectures on PH topics existed in the curriculum. The first-semester Health Systems Sciences (HSS) course included lectures on environmental justice, lead poisoning, and occupational health, but PH themes disappeared from the curriculum after the conclusion of the HSS course.

During early discussions with the PH-TF, the main concern from the Office of Medical Education about improving PH education was the limited time available to cover new topics. To solve this problem, the curriculum working group proposed to integrate PH longitudinally within the existing curriculum. Similar concerns about limited curricular space are widely shared across health professions schools and several other medical institutions have adopted a similar integrative approach to PH education designed to minimally disrupt existing curricula (13–16).

Student members of the PH-TF first reviewed the course objectives for every pre-clerkship course and identified topics that were amenable to being viewed through a PH lens. For each of these topics, students proposed specific PH-related learning objectives that could be addressed within existing lectures. Each proposed learning objective was connected to the relevant PH core competency. A selection of the learning objectives are shown in Table 2.

**Table 2:** Examples of planetary health learning objectives proposed within existing preclinical course objectives.

**Course**	**Course objective**	**PH learning objective**	**PH core competency**
Brain sciences	Pathophysiology of major afflictions of the CNS and PNS	Explain the increased vulnerability to hot weather in patients with chronic neurological conditions such as Parkinson's, MS, and ALS.	Identify the health impacts of climate change and other human-driven disruptions to our natural environment.
Brain sciences	Pathophysiology of common neurologic and psychiatric disorders	Describe the effects on climate change on mental health disorders. Define “climate anxiety” and prepare to speak with a patient about climate anxiety.	Identify the health impacts of climate change and other human-driven disruptions to our natural environment. Apply knowledge of climate and health to clinical care of patients.
Microbiology and infectious disease	Epidemiology and risk factors of infectious diseases	Explain the effects of climate and land use change on vector-borne infections and emerging infectious diseases.	Apply knowledge of the connection between habitat and biodiversity loss and infectious diseases.
Cardiovascular	Risk factors and pathogenesis of common cardiovascular disorders	Identify the role of air pollution in promoting atherosclerosis and cardiovascular disease. Describe the inflammatory cascade produced by air pollution resulting in cardiovascular dysfunction.	Identify the health impacts of climate change and other human-driven disruptions to our natural environment.
Pulmonary	Risk factors and pathogenesis of common pulmonary disorders	Describe the effect that increases in temperature and ambient CO_2_ concentrations has on increasing pollen production and its contribution to allergies. Identify the role that ground-level ozone pollution has on asthma exacerbations, COPD, and pulmonary infections.	Define climate drivers (both natural and human-caused), weather, climate change, and climate variability. Identify the health impacts of climate change and other human-driven disruptions to our natural environment.
Pulmonary	Risk factors and pathogenesis of common pulmonary disorders	Explain the racial and socioeconomic disparities in exposure to air pollution in relation to land use and other policies that disadvantage communities of color.	Recognize the disproportionate impact of climate change on communities of color and explain pre-existing and future health disparities rooted in environmental racism, especially in Rhode Island.
Human reproduction	Risk factors and pathogenesis of common reproductive disorders	Describe the association of air pollution and heat exposure with serious adverse pregnancy outcomes.	Identify the health impacts of climate change and other human-driven disruptions to our natural environment.
Human reproduction	Risk factors and pathogenesis of common reproductive disorders	Identify the disproportionate environmental exposures experienced by pregnant individuals living in underserved communities.	Recognize the disproportionate impact of climate change on communities of color and explain pre-existing and future health disparities rooted in environmental racism, especially in Rhode Island.

The following learning objectives are an illustrative selection of the set of learning objectives proposed by the Planetary Health Task Force to be incorporated into existing pre-clinical courses. Each learning objective falls within the scope of at least one course objective within the existing curriculum. The relevant planetary health core competencies are also presented with each learning objective. Given space constraints, this is not meant to be a comprehensive list of all topic areas within PH.

After the administration accepted this proposal in May 2021, the PH Curriculum Integration Committee (PHCIC) consisting of students, curriculum deans, and faculty experts in PH was formed within the curriculum working group to implement the proposal. The PHCIC's approach to implementation is guided by three principles: efficacy, sustainability, and minimizing disruptions to the existing curriculum. The PHCIC is currently refining the PH learning objectives and planning to incorporate them into courses using an “integration toolbox” which will provide a set format for integration of PH material. Importantly, the PHCIC is focusing on integrating material into case-based small group discussions, as teaching PH through active learning methods has been found to be critical for other institutions' success (15, 16). The PHCIC meets individually with course leaders to introduce the project and solicit feedback, thereby engaging the faculty as stakeholders in this initiative with the goal of promoting sustainability of the initiatives. The PHCIC will also oversee designing an evaluation program to assess impact and enable refinement of the curriculum changes. While this is a multi-year process, the PHCIC plans target a pilot set of the organ systems-based courses for this year's incoming medical students.

#### Education in environmental exposure screening and counseling

**Figure 2:**
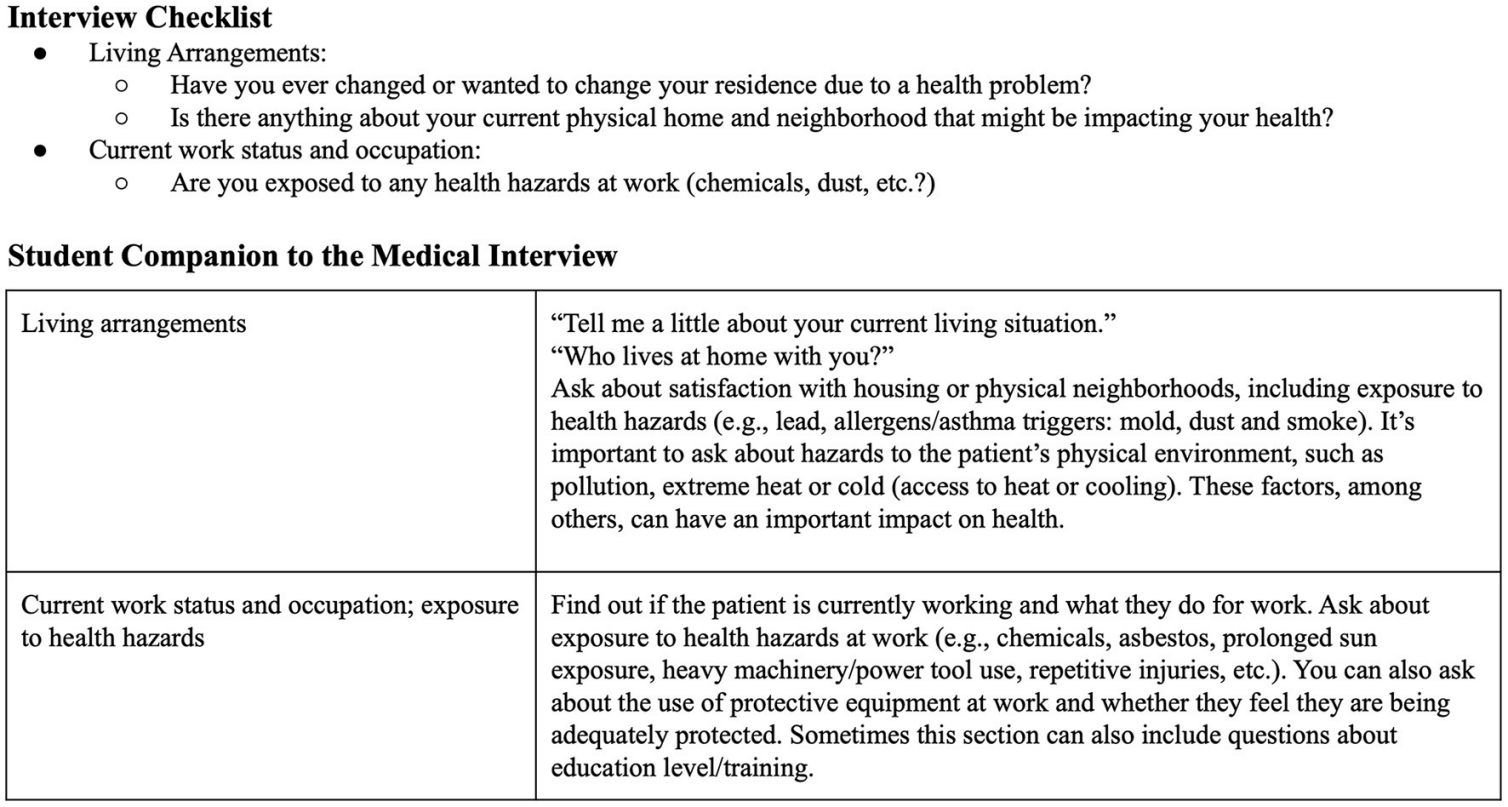
Environmental exposure screening questions incorporated into the social history section of the patient interview checklist taught to first year medical students. Additional context was provided in the Student Companion to the Medical Interview, a medical interview guide available to all students.

Several of the PH core competencies detailed in Table 1 require teaching students to directly address PH issues with affected patients. The PH-TF chose to situate this component of PH education within the school's Doctoring course, a 2-year clinical skills course on interviewing and physical examination skills taught during the pre-clerkship years. To this end, in fall 2021 additional environmental exposure screening questions and context were incorporated into the social history component of the patient interview checklist taught to 1st year medical students. These questions are shown in Figure 2.

In future years, the PH-TF plans to develop a dedicated class on environmental exposure screening and counseling that will be taught to all medical students during Doctoring. The session will include case simulations so students can practice environmental exposure counseling. To provide longitudinal reinforcement of these skills, the PH-TF is also currently in discussions with the family medicine clerkship director about integrating PH screening and counseling within the existing family medicine clerkship curriculum for all 3rd-year medical students.

#### Mandatory waste training for clinical medical students

**Figure 3:**
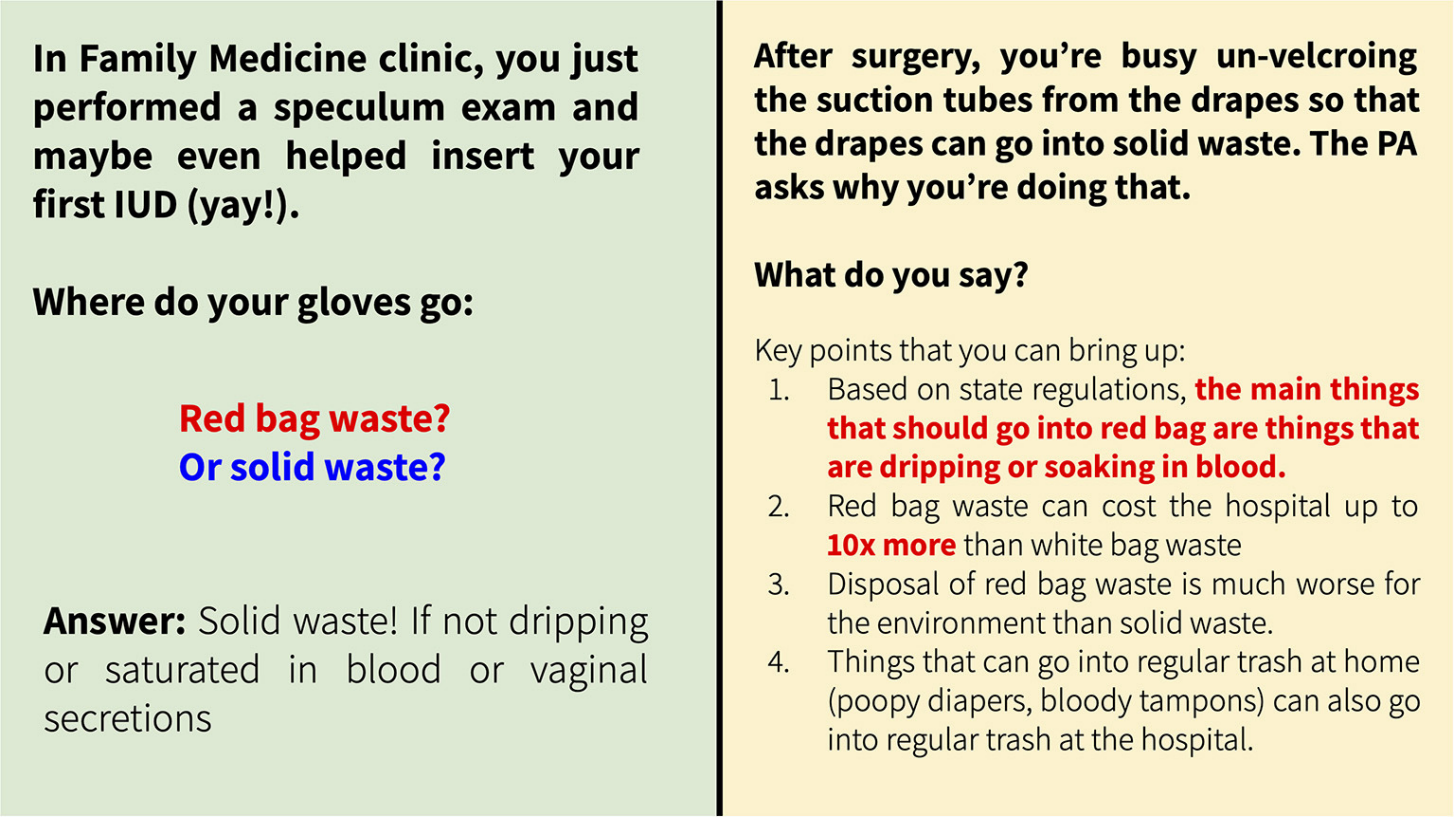
Examples of practice scenarios from the waste disposal training delivered to all medical students entering clerkships.

Prompted by a student-led waste audit at a local hospital, PH-TF students created a mandatory waste training for students entering clerkships. The training is designed to further Ability 8 on moral reasoning and clinical ethics by addressing healthcare impacts on the environment (Table 1), given that the healthcare system produces 10% of US greenhouse gasses and generates four billion pounds of waste each year (6). This training consists of an hour-long session for all 3rd-year medical students during pre-clerkship clinical skills training and has now been taught to two classes of students in 2021 and 2022. It teaches students about proper healthcare waste disposal and decreasing red bag waste, including hospital waste regulations, practice scenarios, and instruction in counseling patients on medical waste disposal at home. Figure 3 shows two practice scenarios from the training.

### Initiatives to facilitate deeper student engagement in planetary health

#### Planetary health elective courses

Although much of the PH curriculum at the Medical School has been designed to reach all medical students, the PH-TF has also created multiple opportunities for students with strong interests in PH to develop additional knowledge and skills. One such opportunity comes *via* PH elective courses. At the Warren Alpert Medical School of Brown University, pre-clerkship electives are offered to 1st- and 2nd-year students. Pre-clerkship electives may be organized by students and either taught by faculty or led by students alongside a faculty advisor. The Medical School also offers electives for clinical students in their third and 4th years, which are typically organized and led by faculty. The PH-TF created a pre-clerkship PH elective that was taught in 2021 and a clinical elective is being developed for the 2022–23 academic year.

Prior to the formation of the PH-TF, a pre-clerkship elective on “Climate Change and Health” was offered to 1st- and 2nd-year medical students in fall 2019. The course exposed students to heat-related morbidity and mortality, changing infectious disease patterns, and the impacts of extreme weather events on human health through lectures, a final project, and community service. Although the COVID-19 pandemic disrupted this elective, the PH-TF revived the elective in fall 2021. Titled Planetary Health: Global Environmental Change and Emerging Infectious Disease, this version of the course took a more focused approach to one facet of PH. Over eight sessions, clinicians, ecologists, and public health experts introduced students to the dynamics of infectious disease emergence resulting from climate change, land-use change, and increased human interaction with wildlife. For a final project, students wrote op-eds about the effects of climate change on human health and were offered guidance to publish their work (17). The elective garnered significant interest in fall 2021 and has been renewed for fall 2022.

PH-TF members are currently crafting a clinical PH elective for 3rd- and 4th-year medical students. This elective's goal is to build on the foundation of pre-clerkship PH knowledge to develop student leaders in PH. This elective will follow an asynchronous curriculum that explores PH education, policy, and clinical impacts. The asynchronous nature of the elective will allow students independence to pursue a specific topic of interest related to education, advocacy, or research within the field of PH under the mentorship of faculty.

#### Facilitating student research in planetary health

Another goal of the PH-TF is to encourage student PH-related research in order to fulfill the PH competencies within Abilities 3 and 7 (see Table 1). Prior to the creation of the PH-TF, small groups of driven medical students had already found and created opportunities to engage in PH research. These efforts have resulted in several publications including waste audits in local hospital emergency rooms and a retrospective study on the impact of summer temperatures on Emergency Medical Services (EMS) utilization (18–21). Ongoing student PH research projects include surveys to quantify the carbon footprint of residency interview travel and Rhode Island EMS and assess hospital food waste as well as a retrospective study on the impact of temperatures at discharge on surgical patient readmissions.

However, a significant challenge for students interested in PH research is the lack of a centralized program devoted to PH research at the Medical School and its affiliated healthcare systems. Medical students currently rely on word-of-mouth to find research mentors with expertise in PH. Compared to other research areas such as sepsis or aging, the Medical School and its affiliated hospital systems have fewer principal investigators engaged in PH research. Recently, the decision of the *Rhode Island Medical Journal* to dedicate an issue to climate change and health helped draw the attention of local researchers and physicians to these issues (22). While hiring new faculty or creating a centralized research initiative dedicated to PH is beyond the scope of the PH-TF, the Medical School can still make progress by directly assisting students interested in PH research as well as drawing the attention of the student body to the potential for scholarship in this area.

To this end, the Medical School administration recently announced a new opportunity for a rising 3rd- or 4th-year medical student to spend a fully funded gap year focused on PH research. The selected student will have the opportunity to sit on the PH-TF and contribute to the task force's initiatives.

In addition, the PH-TF plans to create a new Scholarly Concentration (SC) in Planetary Health. The SC program is a longitudinal commitment to a rigorous independent scholarly project across all 4 years of medical school. Projects are undertaken under the mentorship of a Brown faculty member and further educational and mentorship opportunities are provided by the program directors of each SC. Students choose to participate in the SC program on an elective basis and undergo a competitive application process, with ~25% of the student body selected to participate. As of 2022, there are 12 Scholarly Concentrations at the Medical School, ranging from traditional biomedical research in the Translational Research in Medicine SC to more socially oriented domains such as the Caring for Underserved Communities SC or the Medical Humanities and Ethics SC.

A SC in Planetary Health would serve as a vehicle to consolidate available research opportunities and connect students to relevant faculty. The PH-TF plans to design the SC to help medical students take advantage of resources available in the broader Brown University community by including researchers at Brown's School of Public Health and the Institute at Brown for the Environment and Society. By providing dedicated training in research methods relevant to PH, the SC would enable medical school graduates to advance scholarship in this field throughout their careers.

#### Engaging with and learning from the community

Community engagement is both an important part of medical education and an intrinsic part of the PH movement. While the COVID-19 pandemic negatively impacted many local environmental organizations, the PH-TF's community engagement working group plans to deepen existing partnerships between medical students and the Community Engagement Core of the Brown Superfund Research Program, an initiative that focuses on academic-government-community partnerships to address PH and remediation issues in RI.

## Results to date

The creation of the PH-TF resulted in a more comprehensive, cohesive, and longitudinal PH curriculum. To date a variety of initiatives have been successfully enacted including: (1) various efforts to integrate PH longitudinally within the existing pre-clinical medical curriculum including additions to the medical interview checklist in the Doctoring course and, through the establishment of the PHCIC and resulting interest from course leaders, the addition of a dedicated lecture on air pollution in the 2nd-year Pulmonary course; (2) mandatory waste training for clerkship students to address healthcare impacts on the environment and discuss moral reasoning and clinical ethics; (3) an elective to introduce pre-clerkship students to infectious disease emergence in relation to global environmental change; and (4) the opportunity for a clerkship student to take a fully funded gap year involving PH research and PH-TF initiatives.

The work of the PH-TF is ongoing and the following changes are currently in the process of being implemented: (1) finalization of an integration “toolbox” by the PHCIC with plans to pilot changes for current first-year medical students when they start the organ systems-based courses in spring 2023; (2) creation of a Doctoring session on environmental exposure screening; and (3) a clerkship elective focused on PH policy and clinical impacts. In future years, the PH-TF plans to tackle additional projects including the creation of a PH scholarly concentration for research endeavors and expanding the work of the PH community engagement working group. While no formal assessments of the impact of these initiatives have been completed to date, the PHCIC plans to repeat the PH education needs assessment and use structured surveys to assess the effect of the curriculum changes that will be implemented in the 2022–23 curriculum.

## Discussion of lessons learned and limitations

The approach to PH education at the Warren Alpert Medical School of Brown University has evolved over the past 5 years from a set of sporadic student initiatives into a cohesive structured task force capable of sustainably enacting significant changes over a multi-year time frame. Framing PH learning objectives within the school's established educational priorities, the Nine Abilities, was central to our success because it demonstrated that PH was integral to the mission of the Medical School. Other important factors that led to the success of this initiative include respecting limitations on curricular space by addressing PH topics at their intersection with existing material, creating connections to local environmental and hospital issues, and providing a range of opportunities for both the entire student body as well as a subset of highly interested students. The willingness of the Medical School administration to listen and respond to student concerns has been essential throughout this process.

While we appreciate that each medical school will need to tailor their approaches, we believe that some of the strategies that worked in our context will likely be generalizable to other institutions. Including students, faculty, and administrative members on the PH-TF has substantially hastened the speed with which realistic proposals can be generated and implemented because it enables all parties to communicate directly with each other from the start of the process. The strategy of inserting PH topics within the existing curriculum has succeeded for us and students at several other medical schools because it does not require significant schedule changes. [cite] It also does not overwhelm students with additional lectures nor does it require faculty members to be content experts in PH. The Planetary Health Report Card also served as a helpful starting point for student advocacy efforts that effectively caught the attention of our administration. Finally, ensuring that elective opportunities are available for the subset of students most passionate about PH has helped build relationships between PH-interested students across class years and has effectively created a pipeline for recruiting new student members to the PH-TF, reducing the difficulties associated with student body turnover.

While the curriculum working group of the PH-TF has significantly improved the quality and scope of PH education, the task force structure has its limitations. While the involvement of faculty and administration on the PH-TF has helped provide the continuity required to enact multi-year initiatives, it has still been difficult to build institutional memory about prior PH initiatives given the constant turnover of medical students and the changing schedules of students between pre-clerkship, clerkship, and post-clerkship years. The community engagement working group of the PH-TF has had difficulty making progress because the COVID-19 pandemic disrupted the activities of local environmental organizations for so many months that previous ties between medical students and those organizations were effectively severed when those medical students graduated and moved to residencies without the chance to pass down those connections to the subsequent medical student classes.

In addition, the PH-TF's capacity to enact change has been somewhat limited by the sparsity of medical faculty members with expertise in planetary health education. The lack of a centralized program for planetary health research at the Medical School and its teaching hospitals has also made it difficult to find and recruit new faculty members. While significant research and scholarship related to planetary health occur at the Brown School of Public Health and in the environmental sciences department, the PH-TF has had difficulty coordinating with other parts of the university, in part due to the irregular and sometimes unpredictable hours required during the clinical years of medical school.

Regarding the scope of the PH core competencies, although many of the GCCHE competencies were amenable to being adapted within the framework of the Nine Abilities, some could not be included. For instance, the GCCHE competency “explain the role of subnational, national and global policy frameworks and governance structures to address health risks associated with climate change” were not included because it did not fit easily within the primarily clinical focus of the Nine Abilities. However, while the PH-TF might have difficulty arguing that such topics need to be taught to the entire study body, the elective opportunities created for students with the greatest interest in PH provide flexibility for individual students to pursue such topics if interested.

Finally, while the PH-TF plans to repeat the needs assessment and undertake surveys to assess the impact of PH education initiatives, our conclusions about the effect of the new PH education initiatives on the student body will remain speculative until these structured assessments are completed.

Despite these limitations, we hope our experiences can serve as a useful example for other medical schools interested in implementing their own PH education programs.

## Data availability statement

The original contributions presented in the study are included in the article/supplementary material, further inquiries can be directed to the corresponding author.

## Author contributions

AN-W, BB, JC, KM, and ML conceptualized, wrote, reviewed, and edited the manuscript. AN-W, KM, and JC created figures. SW and KDM reviewed and edited the manuscript. All authors contributed to the article and approved the submitted version.

## Conflict of interest

The authors declare that the research was conducted in the absence of any commercial or financial relationships that could be construed as a potential conflict of interest.

## Publisher's note

All claims expressed in this article are solely those of the authors and do not necessarily represent those of their affiliated organizations, or those of the publisher, the editors and the reviewers. Any product that may be evaluated in this article, or claim that may be made by its manufacturer, is not guaranteed or endorsed by the publisher.

## References

[1] Friedrich M. Medical community gathers steam to tackle climate's health effects. JAMA. (2017) 317:1511–3. 10.1001/jama.2017.0969 28329040

[2] El Omrani O Dafallah A Paniello Castillo B Quintella Ribeiro Correa Amaro B Taneja S Amzil M . Envisioning planetary health in every medical curriculum: an international medical student organization's perspective. Med Teach. (2020) 42:1107–11. 10.1080/0142159X.2020.1796949 32757869

[3] Guzmán CAF Aguirre AA Astle B Barros E Bayles B Chimbari M . A framework to guide planetary health education. Lancet Planetary Health. (2021) 5:e253–5. 10.1016/S2542-5196(21)00110-8 33894134

[4] Maxwell J Blashki G. Teaching about climate change in medical education: an opportunity. J Public Health Res. (2016) 5:673. 10.4081/jphr.2016.673 27190980 PMC4856872

[5] Walpole SC Vyas A Maxwell J Canny BJ Woollard R Wellbery C . Building an environmentally accountable medical curriculum through international collaboration. Med Teach. (2017) 39:1040–50. 10.1080/0142159X.2017.1342031 28681652

[6] Marill M. Pressured by students, medical schools grapple with climate change. Health Aff. (2020) 39:2050–5. 10.1377/hlthaff.2020.01948 33284698

[7] Rabin B Laney E Philipsborn R. The unique role of medical students in catalyzing climate change education. J Med Educ Curric Dev. (2020) 7:1–7. 10.1177/2382120520957653 33134547 PMC7576899

[8] Hampshire K Islam N Kissel B Chase H Gundling K. The Planetary Health Report Card: a student-led initiative to inspire planetary health in medical schools. Lancet Planet Health. (2022) 6:e449–54. 10.1016/S2542-5196(22)00045-6 35461572

[9] Dumenco L George P Scott Taylor J Dollase R. Curriculum innovation at the Warren Alpert Medical School of Brown University. Med Health RI. (2012) 95:317–8, 324–7. 23155850

[10] Wing E Dollase R Dumenco L George P. Innovation and integration at the Warren Alpert Medical School of Brown University. Rev Med. (2016) 95:19. 10.11606/issn.1679-9836.v95ispe3p19-23

[11] Columbia University Global Global Consortium on Climate Health Education. Climate and Health Key Competencies for Health Professions Students. (2019). Available online at: https://www.publichealth.columbia.edu/research/global-consortium-climate-and-health-education/core-competencies-0 (accessed February 23, 2022).

[12] Emont J Bozzi L Monteiro K. Developing a climate change and health curriculum for medical students: a needs assessment. In: Poster Presented at: Annual Meeting of the American Public Health Association. Philadelphia, PA (2019).

[13] Shea B Knowlton K Shaman J. Assessment of climate-health curricula at international health professions schools. JAMA Network Open. (2020) 3:e206609–e206609. 10.1001/jamanetworkopen.2020.6609 32463471 PMC7256668

[14] Sullivan JK Lowe KE Gordon IO Colbert CY Salas RN Bernstein A . Climate change and medical education. Acad Med. (2022) 97:188–92. 10.1097/ACM.0000000000004376 34432714

[15] Kligler SK Clark L Cayon C Prescott N Gregory JK Sheffield PE. Climate change curriculum infusion project: An educational initiative at one US medical school. J Clim Change Health. (2021) 4:100065. 10.1016/j.joclim.2021.100065

[16] Kligler B Pinto Zipp G Rocchetti C Secic M Ihde ES. The impact of integrating environmental health into medical school curricula: a survey-based study. BMC Med Educ. (2021) 21. 10.1186/s12909-020-02458-x 33419439 PMC7796639

[17] Picard K. Why Doctors Should Advocate for More Green Space. Planetary Health Alliance/Medium (2022). Available online at: https://phalliance.medium.com/why-doctors-should-advocate-for-more-green-space-d43ba6130ac1 (accessed February 23, 2022).

[18] Denison Martin K Chen JJ Thorndike J McCormick W Rota J Berg B . Trends in waste production at a community hospital during the COVID-19 pandemic. Rhode Island Med J. (2021) 104:38–42. 34705906

[19] Hsu S Banskota S McCormick W Capacci J Bustamante C Moretti K . Utilization of a waste audit at a community hospital emergency department to quantify waste production and estimate environmental impact. J Clim Change Health. (2021) 4:100041. 10.1016/j.joclim.2021.100041

[20] Hsu S Thiel CL Mello MJ Slutzman JE. Dumpster diving in the emergency department: quantity and characteristics of waste at a level I trauma center. West J Emerg Med Integr Emerg Care Popul Health. (2020) 21:1211–7. 10.5811/westjem.2020.6.47900 32970577 PMC7514403

[21] Moretti K Gallo Marin B Soliman L Asselin N Aluisio A. Increased temperatures are associated with increased utilization of Emergency Medical Services in Rhode Island. Rhode Island Med J. (2021) 104:24–8. 34705903

[22] Binder W. Climate change and health: a special edition of the Rhode Island Medical Journal. Rhode Island Med J. (2021) 104:9. 34705898

